# The role of artificial intelligence in postoperative clinical decision-making for pancreatic cancer: a pilot study

**DOI:** 10.3389/fsurg.2026.1802926

**Published:** 2026-06-01

**Authors:** Samet Yigman, Ahmet Onur Demirel, Ibrahim Halil Ozata, Burak Çelik, Safa Toprak, Salih Karahan, Volkan Adsay, Orhan Bilge, Gürkan Tellioğlu

**Affiliations:** 1 Department of General Surgery, Koç University School of Medicine, Istanbul, Türkiye; 2 Department of Pathology, Koç University School of Medicine, Istanbul, Türkiye

**Keywords:** artificial intelligence, clinical decision support, decision-making, multidisciplinary tumor board, pancreatic cancer

## Abstract

**Objective:**

Postoperative follow-up and treatment decisions after pancreatic cancer surgery are routinely determined by multidisciplinary tumor boards (MDTs), which represent the mainstay of clinical decision-making in these patients. However, this process is associated with increased workload, time consumption, and financial burden. Previous studies have demonstrated improved treatment planning, guideline adherence, and patient outcomes with MDTs, albeit with substantial time, cost, and administrative demands. The present study aims to compare MDT-based decision-making with an artificial intelligence (AI)–assisted model in the postoperative management of patients undergoing surgery for pancreatic cancer and to explore the potential utility of such a model.

**Materials and methods:**

An AI-based model was developed using clinical data from 67 patients discussed in multidisciplinary tumor boards between October 2020 and December 2023, in conjunction with current treatment guidelines. The model's recommendations were subsequently compared with MDT decisions in an independent cohort of 15 patients who underwent surgery in 2024.

**Results:**

The overall concordance rate between AI-generated recommendations and MDT decisions was 80%. The Cohen's kappa coefficient was 0.625 (95% CI: 0.278–0.972), indicating moderate agreement beyond chance. Three discrepant cases were further analyzed to explore potential reasons for discordance.

**Discussion:**

The findings suggest that AI-assisted decision-support systems may approximate MDT recommendations in postoperative pancreatic cancer management. However, observed discrepancies highlight the continued importance of expert clinical judgment and contextual interpretation in complex decision-making scenarios.

**Conclusion:**

AI-based models may serve as supportive tools in postoperative clinical decision-making by potentially reducing workload and time burden, but should complement rather than replace multidisciplinary expert evaluation.

## Introduction

### Background and significance

Pancreatic cancers, particularly pancreatic ductal adenocarcinoma, are often diagnosed at advanced stages and are characterized by an aggressive biological behavior, resulting in dismal outcomes. Although pancreatic cancer ranks as the 14th most commonly diagnosed cancer worldwide, it represents the 7th leading cause of cancer-related mortality (1). Because a substantial proportion of patients present with locally advanced or metastatic disease at the time of diagnosis, treatment remains challenging and survival outcomes are poor. Consequently, the determination of optimal postoperative follow-up and treatment strategies is of critical importance.

Postoperative management decisions are traditionally determined through multidisciplinary tumor boards (MDTs), in which patient- and disease-specific factors are evaluated by physicians from multiple specialties, including oncology, surgery, pathology, radiology, nuclear medicine, and radiation oncology. While MDT meetings provide an individualized and guideline-informed framework for patient management, they are not without limitations (2). The organization of these meetings requires the regular coordination of physicians from different departments and involves additional processes such as case preparation, presentation, meeting organization, and administrative support. As a result, MDTs may lead to considerable time consumption, increased workload, and financial burden. Particularly in high-volume centers, these demands may further increase clinicians' workload, limit the time available for academic and clinical activities, and adversely affect overall productivity (3). These challenges underscore the need for more efficient and supportive clinical decision-making mechanisms.

Artificial intelligence (AI) algorithms have emerged as valuable tools in clinical decision-making due to their ability to rapidly analyze large datasets, identify complex patterns, and interpret unstructured information such as data derived from the medical literature. With recent advances in AI technology, their potential role in clinical diagnostic and therapeutic decision support has gained increasing attention. Previous studies have demonstrated that large language model-based and other AI-based systems may improve diagnostic and treatment accuracy, reduce case evaluation time, and enhance adherence to clinical guidelines across various disease settings (4, 5).

In the present study, we aimed to develop an accessible artificial intelligence–assisted decision support model that is easily accessible and capable of natural language processing and complex data analysis, and to evaluate its feasibility in supporting postoperative follow-up and treatment decision-making in patients undergoing surgery for pancreatic cancer.

## Materials and methods

### Ethical approval and study design

This study was approved by the Koç University School of Medicine Ethics Committee under protocol number 2025.017.IRB2.013. An artificial intelligence–based decision support model utilizing a large language model architecture (ChatGPT-4.0, OpenAI) was developed to support postoperative follow-up and treatment decision-making in patients undergoing surgery for pancreatic ductal adenocarcinoma (PDAC), and its recommendations were compared with those of the multidisciplinary tumor board (MDT).

The study design incorporated retrospective data, with patient records between October 2020 and December 2023 used for model development and contextual grounding, and data from 2024 used to form an independent comparison cohort.

### Development of the AI model

The AI model was based on the ChatGPT-4.0 large language model (OpenAI). No fine-tuning or retraining was performed; instead, a prompt-based approach was employed. During model development, anonymized clinical data from 67 patients with PDAC who underwent surgical resection and were discussed in multidisciplinary tumor boards at the Department of General Surgery, Koç University School of Medicine between October 2020 and December 2023 were used to inform the clinical context of the model.

The clinical variables used for model development included the American Society of Anesthesiologists (ASA) score, comorbidities, neoadjuvant treatment status, TNM staging, and detailed pathological findings, including tumor location, surgical margin status, total number of harvested lymph nodes, number of metastatic lymph nodes, presence of invasion into surrounding tissues or vascular structures, and whether vascular resection was performed.

For each patient, the model was prompted to recommend one of the following postoperative management strategies: follow-up, adjuvant chemotherapy, or adjuvant chemoradiotherapy. All patient data were entered into the model in a standardized and structured format, and a single predefined prompt template was used consistently across all cases without modification. The model generated a single recommendation per case, and no iterative prompting, prompt engineering variations, or response selection processes were applied. Model parameters (including temperature) were maintained at default settings throughout the study.

To minimize circularity, MDT decisions were not provided to the model as input during the validation phase. MDT-derived insights and NCCN guideline-based principles were incorporated only at the prompt design stage as conceptual frameworks, and were not used as direct inputs or training targets. During evaluation, the model received only patient-specific clinical and pathological variables and independently generated recommendations.

Importantly, the model was not instructed to replicate MDT decisions, but rather to generate independent recommendations based on structured clinical inputs. Instead, it was prompted to integrate all available clinical and pathological information within a guideline-informed context. This approach was intentionally designed to minimize operator-dependent variability, reduce prompt-induced bias, and ensure that the outputs reflect the model's intrinsic decision-making behavior rather than *post hoc* prompt optimization.

The exact prompt used in this study is provided in the Supplementary Material (Supplementary Material S1) to ensure transparency and reproducibility.

An AI-based model was selected due to its widespread accessibility and its capacity for natural language processing, enabling effective interpretation of unstructured clinical data such as narrative pathology reports and supplementary clinical notes. After patient-specific clinical features were defined within the ChatGPT interface, the model was asked to generate a postoperative follow-up or treatment recommendation.

### Patient selection

All patients who underwent surgical resection for PDAC were eligible for inclusion, regardless of the type of surgical procedure, neoadjuvant treatment status, or disease stage. This inclusive approach was intentionally adopted to reflect the real-world clinical heterogeneity encountered in multidisciplinary tumor boards.

### Statistical analysis

Given the pilot and exploratory nature of this study, no hypothesis-driven inferential statistical analyses were performed. The primary objective was to evaluate the level of agreement between MDT recommendations and the AI-based model. Agreement was assessed using descriptive concordance rates. In addition, Cohen's kappa coefficient was calculated as an exploratory measure of agreement beyond chance. Statistical analyses were performed using SPSS version 28. Due to the limited sample size, no inferential statistical testing was planned.

### Comparison and evaluation

To evaluate the performance of the AI model, 15 patients who underwent surgical resection in 2024 and were discussed in multidisciplinary tumor boards were included as the comparison cohort. The clinical data of these patients were provided to the AI model, which was asked to generate postoperative follow-up or treatment recommendations. These recommendations were subsequently compared with MDT decisions.

Agreement and discrepancies between AI-generated recommendations and MDT decisions were analyzed on a patient-by-patient basis, and potential reasons for discordance were explored. This approach aimed to identify not only the strengths of the AI model but also areas requiring further refinement. In particular, the extent to which patient-specific factors—such as comorbidities, overall health status, and functional condition—were incorporated by the AI model and influenced its decision-making process was evaluated.

## Results

### Concordance rate of the AI model

When the 15-patient validation cohort was evaluated, the overall concordance rate between MDT recommendations and the AI-based model was 80% (12 of 15 cases). Detailed patient characteristics and the corresponding MDT and AI-based recommendations are summarized in Table 1. Exploratory agreement analysis yielded a Cohen's kappa value of 0.625 (95% CI: 0.278–0.972), indicating moderate agreement beyond chance, with wide confidence intervals reflecting the limited sample size.

**Table 1:** Detailed patient characteristics and the corresponding MDT and AI-based recommendations.

Patient No	Age-Sex	Neoadjuvant Treatment	Operation	TNM	Additional Findings	MDT Recommendation	AI Recommendation
1	60/M	No	Whipple procedure	T2N2	vascular resection performed and surgical margin positive	CRT	CRT
2*	61/M	Yes	Whipple procedure	T1N0	extends to vascular bed	Follow-up	CT
3*	81/M	No	Subtotal pancreatectomy	T3N2	perinodal and adipose tissue invasion	CT	CRT
4	84/M	No	Whipple procedure	T2N1	perinodal and adipose tissue invasion, surgical margin <1mm	CRT	CRT
5	68/M	Yes	Total pancreatectomy	T2N2	vascular resection performed, perinodal invasion	CRT	CRT
6	70/F	No	Subtotal pancreatectomy	T2N1	perinodal invasion	CT	CT
7	82/F	Yes	Whipple procedure	T1N0M1	liver metastasis	CT	CT
8	47/M	No	Whipple procedure	T3N2	surgical margin <1 mm	CRT	CRT
9	67/M	No	Whipple procedure	T3N1	perinodal invasion, vascular resection performed	CT	CT
10	51/F	Yes	Subtotal pancreatectomy	T2N2	-	CT	CT
11*	76/M	No	Total pancreatectomy	T3N1	vascular resection performed	CT	CRT
12	52/M	No	Whipple procedure	T2N2	extends to peripancreatic and retroampullar area	CRT	CRT
13	32/F	No	Whipple procedure	T3N0	-	CT	CT
14	71/F	No	Total pancreatectomy	T2N2	-	CRT	CRT
15	59/M	No	Whipple procedure	T3N1	surgical margin positive	CRT	CRT

MDT, multidisciplinary tumor board; CT, chemotherapy; CRT, chemoradiotherapy.

### Analysis of discrepant cases

The three discrepant cases (Patients 2, 3, and 11) were examined in detail with respect to patient characteristics, MDT and AI-based recommendations, potential reasons for discordance, and clinical outcomes.

1. **Case 2: Adjuvant Treatment Discrepancy**
  61-year-old male patient underwent pancreaticoduodenectomy for a pancreatic head tumor following neoadjuvant therapy. Histopathological evaluation revealed a T1N0-stage tumor with negative surgical margins; however, tumor extension toward vascular structures was observed, and lymphatic, vascular, and perineural invasion were present. The tumor was classified as low-grade.
  Taking the patient's comorbidities and prior neoadjuvant treatment into account, the MDT recommended postoperative surveillance. In contrast, the AI-based model recommended adjuvant chemotherapy. During follow-up, recurrence was detected in the peripancreatic region.
2. **Case 3: Adjuvant Treatment Discrepancy**
  An 81-year-old male patient underwent upfront subtotal pancreatectomy for a pancreatic body tumor. Pathological examination demonstrated T3N2-stage tumor with extension into the duodenal mucosa. Surgical margins were negative, while lymphatic, vascular, and perineural invasion were positive, and the tumor was classified as low-grade.
  Considering the patient's advanced age and comorbidities, the MDT favored a less intensive systemic treatment approach. Conversely, the AI-based model proposed a more comprehensive treatment strategy, including the addition of radiotherapy. At one-year follow-up, the patient remained on systemic therapy, and no evidence of recurrence was observed.
3. **Case 11: No Treatment Recommendation and Recurrence**
  76-year-old male patient underwent total pancreatectomy following upfront surgery for a pancreatic body tumor. The tumor was staged as T3N1, with extension into the vascular resection area. Surgical margins were negative, and lymphatic, vascular, and perineural invasion were present. The tumor was classified as low-grade.
  The MDT recommended adjuvant chemotherapy after considering the patient's overall condition and individual clinical factors. The AI-based model initially suggested combined chemotherapy and radiotherapy, but additionally noted that if the patient's general condition was insufficient to tolerate combined therapy, prioritization of adjuvant chemotherapy should be considered. As postoperative follow-up was conducted in another city, outcome data were not available for this patient.

## Discussion

In this study, the performance of an AI-based model in determining postoperative follow-up and treatment decisions for patients with PDAC was evaluated, and differences between AI-generated recommendations and current clinical practice based on multidisciplinary tumor boards (MDTs) were examined. The heterogeneity of the patient cohort represents a deliberate design choice aimed at assessing the robustness of AI-based recommendations across diverse clinical scenarios rather than an attempt to demonstrate superiority over MDT decisions.

One of the most notable findings of this study is the 80% concordance rate between MDT decisions and recommendations generated by the AI-based model. The observed Cohen's kappa value of 0.625 suggests that, despite the clinical heterogeneity of the cohort and the limited sample size, the agreement between MDT and AI-based recommendations extends beyond simple concordance and reflects a meaningful level of consistency. However, the wide confidence interval associated with the kappa estimate reflects the limited sample size and indicates uncertainty in the strength of agreement. Therefore, these findings should be interpreted cautiously. These findings suggest that the AI model may be capable of analyzing complex clinical scenarios and generating clinically relevant recommendations. However, given the exploratory nature of this pilot study, these findings should not be interpreted as definitive evidence of performance, but rather as an initial indication of feasibility that requires validation in larger and more diverse cohorts.

Moreover, these findings highlight the potential of AI-based models to substantially reduce clinician workload and accelerate decision-making processes, particularly in standard clinical scenarios where patient-specific qualitative factors play a limited role. Given the time- and resource-intensive nature of MDT meetings, integrating AI models into this process may improve efficiency, reduce administrative burden, and allow clinicians to focus more effectively on complex or borderline cases.

Further insights emerge from the analysis of discrepant cases, which are summarized in Table 2. These differences reveal both the limitations of the AI model and, in certain contexts, its potential strengths. In two cases, MDT decisions favored less intensive treatment strategies after carefully considering patient-specific factors such as age, comorbidities, and overall functional status, reflecting a patient-centered approach that balances survival benefit with treatment tolerance and quality of life. This approach integrates clinical experience and prior treatment responses to optimize outcomes within an acceptable therapeutic burden.

**Table 2:** Detailed analysis of discordant cases between MDT and AI-based recommendations.

Patient No	Key Clinical Features	MDT vs. AI	Reason for Discordance	Clinical Outcome/Notes
2	61/M, neoadjuvant therapy, T1N0, vascular proximity	Follow-up vs. CT	MDT favored conservative approach due to prior treatment and comorbidities; AI prioritized pathological risk factors	Peripancreatic recurrence observed
3	81/M, T3N2, extensive invasion, elderly with comorbidities	CT vs. CRT	MDT influenced by advanced age and performance status; AI recommended intensified therapy based on tumor burden	No recurrence at 1-year follow-up
11	76/M, T3N1, vascular involvement	CT vs. CRT (conditional)	AI incorporated conditional recommendation based on tolerance; MDT opted for standard chemotherapy	Outcome not available

In one discrepant case, recurrence was observed during follow-up. However, this observation should be interpreted with caution, as recurrence is a multifactorial outcome and cannot be directly attributed to the appropriateness of a single treatment recommendation. This finding underscores the complexity of clinical decision-making in this setting.

Although the AI model was informed by retrospective clinical data and established treatment guidelines, it remains limited in its ability to fully capture abstract and individualized factors such as patient preferences, expected quality of life, treatment-related toxicity, short- and long-term expectations, and the relative importance of prognostic variables for individual patients. Therefore, AI models should not be viewed as standalone decision-makers but rather as complementary tools that support and validate MDT-based clinical decision-making. In this context, clinical experience, physician judgment, and the physician–patient relationship remain indispensable components of the decision-making process.

Conversely, by focusing exclusively on guideline-based evidence and data-driven analysis—without being influenced by emotional or cognitive biases—AI models may have potential in identifying patients at high risk of recurrence and highlighting potentially beneficial treatment strategies. This capability supports the use of AI as a clinical decision-support tool that enriches, rather than replaces, existing MDT processes. A hybrid approach combining the analytical speed and consistency of AI with human insight, intuition, and empathy may offer the most balanced and effective strategy for clinical decision-making.

An important factor underlying the differences observed between multidisciplinary tumor board (MDT) decisions and artificial intelligence (AI)–based recommendations is the contribution of experienced expert opinion that extends beyond quantitatively measurable clinical variables. In routine clinical practice, treatment decisions are not always based solely on formal staging parameters or guideline-defined pathological features. In particular, experienced pathologists may take into account qualitative assessments derived from microscopic patterns, tumor architecture, or atypical biological behavior that are not explicitly stated in standard pathology reports. Such expert-level interpretations, grounded in accumulated clinical experience, may lead MDTs to favor either more intensive or more conservative treatment strategies, even when quantitative data are otherwise similar. In contrast, AI-based models inherently rely on structured inputs and guideline-driven parameters. This distinction highlights a fundamental difference between human-led clinical reasoning and algorithmic decision-support systems, and represents an important source of divergence between MDT and AI recommendations.

From a methodological perspective, it is important to note that, although MDT practices and guideline-based principles informed the conceptual design of the model, MDT decisions were not provided to the model as input during evaluation. Therefore, the observed agreement does not reflect simple replication of MDT reasoning, but rather an independent generation of recommendations based on structured clinical inputs.

In addition, AI-based models may serve as objective, evidence-based tools for assessing decision accuracy within and across institutions. This could facilitate standardization of clinical decision-making processes, reduce unwarranted variability, and enable longitudinal monitoring of decision quality based on literature-supported metrics. Such an approach may enhance MDT effectiveness and contribute valuable data for future educational initiatives and quality improvement programs.

This study has several limitations, including its single-center design and the relatively small number of patients in both the training and comparison cohorts. In addition, MDT decisions were used as a pragmatic real-world comparator rather than an absolute reference standard, as such decisions are inherently context-dependent, influenced by institutional practices, and may vary across clinical settings. The performance of the AI model is expected to improve with access to larger, more diverse datasets and more granular guideline specifications. Future iterations incorporating patient-specific variables such as quality of life, treatment-related toxicity, adherence, and long-term survival outcomes may further enhance the model's decision-making capacity. Multicenter studies with larger cohorts will be essential to validate the generalizability and reliability of AI-based models, as well as their potential for iterative refinement with larger datasets. Integration of such systems into routine clinical practice will also require careful consideration of ethical and legal implications. Nevertheless, in selected patient populations—such as younger patients without significant comorbidities—AI-based decision support may demonstrate higher concordance and be associated with fewer ethical or legal concerns.

The relatively small size of the validation cohort represents an important limitation, as it may not fully reflect the complexity and variability of postoperative clinical decision-making in pancreatic cancer. Accordingly, the findings should be interpreted as preliminary and hypothesis-generating.

## Conclusion

This study suggests that an AI-based model shows a high level of concordance with multidisciplinary tumor board decisions in the postoperative management of pancreatic ductal adenocarcinoma. These findings suggest that AI-based models may serve as supportive clinical decision-making tools with the potential to reduce clinical workload.

## Data Availability

The original contributions presented in the study are included in the article/Supplementary Material, further inquiries can be directed to the corresponding author.
